# Awareness of Climate Change and the Dietary Choices of Young Adults in Finland: A Population-Based Cross-Sectional Study

**DOI:** 10.1371/journal.pone.0097480

**Published:** 2014-05-13

**Authors:** Essi A. E. Korkala, Timo T. Hugg, Jouni J. K. Jaakkola

**Affiliations:** 1 Center for Environmental and Respiratory Health Research, University of Oulu, Oulu, Finland; 2 Medical Research Center Oulu, Oulu University Hospital and University of Oulu, Oulu, Finland; 3 Public Health, Institute of Health Sciences, University of Oulu, Oulu, Finland; Faculty of Medicine & Allied Sciences, Rajarata Univeresity of Sri Lanka, Sri Lanka

## Abstract

Climate change is a major public health threat that is exacerbated by food production. Food items differ substantially in the amount of greenhouse gases their production generates and therefore individuals, if willing, can mitigate climate change through dietary choices. We conducted a population-based cross-sectional study to assess if the understanding of climate change, concern over climate change or socio-economic characteristics are reflected in the frequencies of climate-friendly food choices. The study population comprised 1623 young adults in Finland who returned a self-administered questionnaire (response rate 64.0%). We constructed a Climate-Friendly Diet Score (CFDS) ranging theoretically from −14 to 14 based on the consumption of 14 food items. A higher CFDS indicated a climate-friendlier diet. Multivariate linear regression analyses on the determinants of CFDS revealed that medium concern raised CFDS on average by 0.51 points (95% confidence interval (CI) 0.03, 0.98) and high concern by 1.30 points (95% CI 0.80, 1.80) compared to low concern. Understanding had no effect on CFDS on its own. Female gender raised CFDS by 1.92 (95% CI 1.59, 2.25). Unemployment decreased CFDS by 0.92 (95% CI −1.68, −0.15). Separate analyses of genders revealed that high concern over climate change brought about a greater increase in CFDS in females than in males. Good understanding of climate change was weakly connected to climate-friendly diet among females only. Our results indicate that increasing awareness of climate change could lead to increased consumption of climate-friendly food, reduction in GHG emissions, and thus climate change mitigation.

## Introduction

Climate change has been characterized as the biggest global health threat of the 21^st^ century [1]. The probable adverse health effects of climate change include more daily deaths due to temperature extremes, increased allergic disorders due to longer pollen season and increased risk of infectious disease due to flooding [2]. By mitigating climate change we can promote public health in the future.

From the public health perspective it is beneficial to promote especially those climate change mitigation actions that are good for health directly. Climate-friendly food consumption is an example of such behavior: through certain dietary choices one can mitigate climate change and promote his or her own health at the same time [3], [4]. This is because adjusting into a more climate-friendly diet decreases the risk of many diseases such as coronary heart disease and cancer [3]–[5]. For these reasons encouraging climate-friendly eating is reasonable from the public health perspective.

Climate change and food production are closely connected. The production of food is a major contributor to anthropogenic greenhouse gas (GHG) emissions which are the most important cause of climate change [6]. Worldwide the agriculture sector is responsible for 22% of total GHG emissions [7] and together with food processing it causes approximately one-third of total GHG emissions [8].

Food products differ substantially in the amount of GHGs their production generates. The specific food items associated with high GHG emissions include beef, sheep, pork, cheese, rice and butter [9]–[11]. On the other hand food items like fresh vegetables, potatoes and margarine are associated with low GHG emissions [9]–[12]. In Finland, the typical diet of an adult (aged 25 to 74) is high in both climate-friendly and non-climate-friendly food items [13]. For example, majority of Finnish adults consume potatoes rather than rice as a side dish, which is a climate-friendly choice. On the other hand, many non-climate friendly food items such as red meat and cheese are also consumed by the majority of adults in Finland. Reducing the consumption of foods that are associated with high GHG emissions is a feasible and practical way to mitigate climate change [7], [14], [15]. Therefore predictors of climate-friendly food choices are of interest.

There is some research on the Finnish people’s perceptions about climate change. According to the Eurobarometer 2008 [16], 78% of Finnish adults perceive climate change as a very serious problem. 73% of Finnish people agree that climate change is among the two biggest global problems at the moment. Therefore it seems that the public in Finland is aware of climate change and perceives climate change as a serious threat. Finnish people recognize the link between climate change and different consumption behaviors quite well [17]. For example, 93% of Finns agree that reducing car use would have quite a big effect or a big effect on climate change. More than 80% agree that residential heating, travelling and electricity consumption have quite a big effect or a big effect on climate change. In addition, 42% of Finnish adults agree that favoring a plant-based diet has quite a big effect or a big effect on climate change [17].

There is only a limited number of previous literature on the predictors of climate-friendly eating [18], [19]. The perceived seriousness of climate change consequences seems to be a predictor of climate-friendly food choices among social science university students [18]. Pro-environmental self-identity has been found to predict climate-friendly shopping and eating [19]. It is not known if the determinants of climate-friendly eating differ between the genders or different socio-economic groups. In addition, previous studies base their assessment of climate-friendliness of a diet on only a few measures rather than diet-wide assessment of the intake frequencies of different food items [18], [19]. Therefore there is a need for information on the determinants of climate-friendly eating across different socio-economic groups and genders with assessment of actual food intake frequencies. To add to the knowledge on the predictors of climate-friendly food choices, we conducted a study among young adults in Finland. Our primary aim was to assess if understanding of and concern over climate change are reflected in the frequencies of climate-friendly food choices. In addition, we studied the role of gender and socio-economic factors as determinants of climate-friendly food choices.

We hypothesized that people with high concern over climate change make climate-friendlier dietary choices than people who are not concerned over climate change. This hypothesis was based on the previous finding that high concern over climate change predicts mitigation actions [20], [21]. We also hypothesized that good understanding of climate change is connected to climate-friendlier dietary choices since heightened knowledge about environmental problems is associated with pro-environmental behaviors [22]. However, we expected the effect of understanding to be smaller than that of concern because people’s environmental behavior is not always in accordance with their knowledge [19], [23]. Our hypothesis concerning the socio-demographics was that female gender and high educational level are connected to climate-friendly eating since these two factors have been found to be predictors of climate change mitigation action [21], [24], [25].

## Methods

### Study Population

This was a population-based cross-sectional study. The study population was the Espoo cohort established in 1991 when the cohort members were living in the city of Espoo in Southern Finland. The cohort consists of 2568 members born between January, 1984 and March, 1990. For this 20-year follow-up the contact information of the cohort members was acquired from the Population Register Centre (The Population Register Centre operates under the Ministry of Finance and contains basic identification information about all Finnish citizens) [26]. A self-administered, multiple choice questionnaire was sent to 2534 cohort members whose address was available between March 2010 and June 2011. The information gathering consisted of several posting rounds as well as phone contacts. 1623 completed questionnaires were received (response rate 64.0%). The respondents were a representative sample of the original baseline study population as reported in another study on the 20-year follow up [27]. The questionnaire contained several sections and was partly based on questions used in the previous follow-ups and research projects [28], [29]. The study was approved by the Ethics Committee of the Oulu University Hospital District.

### Assessment of Awareness of Climate Change

Awareness of climate change was evaluated by assessing understanding of climate change and concern over climate change. Assessment of understanding of climate change was based on the question: *What do you think is meant by climate change?* Respondents were to choose their preferred definition of climate change from the following five definitions: *Global warming of the climate caused by 1 an increase in sunspot activity and sun’s radiant energy*, *2 a change in the axial tilt of the Earth*, *3 an increase in population growth, energy consumption and exploitation of nature*, *4 an increase in the greenhouse gas concentration of the atmosphere derived from human actions* and *5 a natural fluctuation of climate periods on Earth*. We judged alternatives 3 and 4 to represent good understanding and alternatives 1, 2 and 5 poor understanding of climate change. This judgment was based on the causes of climate change as reported by the Intergovernmental Panel on Climate Change [6].

The degree of concern over climate change was assessed on the basis of the answer to the question: *If the climate is in some way changing, how a serious threat to the humankind do you think it is?* The five alternatives were: *1 A very great threat*, *2 Quite a great threat*, *3 Not a special threat*, *4 Not a threat at all* and *5 I do not know*. Alternative 1 indicated high concern, alternative 2 medium concern and the rest low concern over climate change.

### Assessment of Dietary Choices

The food consumption during the past 12 months was assessed by asking the intake frequency of food items on a 5-point scale (*less than once a month*, *1–3 times a month*, *1–3 times a week*, *almost daily*, *at least once a day*). The intake frequency of organic food was asked on a different scale (*not at all, less frequently than once a month, 1–3 times a month, 1–3 times a week, daily or almost daily*). For the analysis we selected the food items especially climate-friendly and the ones non-climate-friendly. The climate-friendliness of a food item was defined by the GHG emissions created by the production of the food item from farm to table (as measured in CO_2_ equivalents per 1 kg of food produced). The emissions of different food items were acquired from the literature [9], [10], [29]–[32] and compared to make the distinction between climate-friendly and non-climate friendly food items. We used emission data from European studies, mainly from Sweden, where the conditions are comparable with those in Finland. In the case of French fries information on energy consumption during production and preparation was used as an indicator of climate-friendliness [12] because information on GHG emissions was not available for this food item. Specific information on the GHGs emitted by the production of soy products was not available, so a general value for meat substitutes (tofu, tempeh, lupin and vegaburgers) was judged to apply to soy products [30]. In our study, climate-friendly food items included fresh vegetables/salad/root vegetables [10], [31], soy products (such as tofu) [30], potatoes (cooked or mashed) [10], fresh fruits [10], margarines [11], vegetable oils [10] and organic food [32], [33]. Non-climate-friendly food items included pork/beef/lamb [10], [31], poultry [10], low fat cheese [10], other cheese [10], rice [10], butter [11] and French fries [12]. The individual intake frequencies of the food items were compared with the median intake frequency of the study population and were classified as high (>median), average and low (<median).

To assess the overall climate-friendliness of the respondents’ diets, a novel measure, the climate-friendly diet score (CFDS) was generated. CFDS was constructed to be a comparative measure which uses the typical diet of a Finnish adult as the baseline. CFDS was calculated for each respondent based on how often they consume the food products in consideration. One point was given for high frequency intake of the climate-friendly food items whereas one minus point was given for low intake of these items. One minus point was given for high frequency intake of non-climate-friendly food items and one plus point for low frequency intake of these items. No points were given if the intake of the food item in question was average as this was judged to indicate no dietary adjustment into any direction. CFDS was calculated as a sum of the points given. Thus the theoretical range of the CFDS was from −14 to 14 and a higher score indicated a climate-friendlier diet.

### Statistical Methods

The relations of interest were 1) the level of understanding of climate change and the consumption of climate-friendly food and 2) the level of concern about climate change and the consumption of climate-friendly food and 3) the socio-demographic factors and the consumption of climate-friendly food.

First, the intake frequencies of selected items were compared according to understanding (poor vs. good) and concern (low, medium and high). The role of chance in the differences between the frequency distributions was assessed applying Chi square-test and corresponding trend test. Second, the average CFDS’s were compared between categories of understanding and concern of climate change. Finally, the determinants of CFDS were modeled with multivariate linear regression analysis using the following variables: understanding of climate change, concern over climate change, gender, education, occupation, marital status, parental status and average annual income. The presence of interaction between understanding and concern was assessed by fitting corresponding product terms (understanding*high concern) in addition to actual variables for independent effects. Weak positive interaction was observed but it turned out not to be statistically significant. The statistical software used for all analyses was SAS 9.3.

## Results

### Characteristics of the Study Population

Out of the whole study population (n = 1623) 89.3% had good understanding and 10.7% poor understanding of climate change. 35.6% had high concern over climate change, 47.5% medium concern and 16.9% low concern.

Women had on average better understanding and higher concern over climate change compared to men (Table 1). Age within this narrow range did not seem to have a clear effect on understanding nor concern. Higher vocational or academic degree holders were highly concerned about climate change whereas comprehensive school and vocational school degree holders were underrepresented in the highly concerned. Understanding of climate change was fairly good in all educational groups except vocational school degree holders, who were overrepresented among the people who understand climate change poorly. The unemployed and people working in the factory, mining or construction trade tended to have low concern over climate change. Marital status or the presence of children did not seem to affect understanding of or concern over climate change. People in the highest income category were less concerned about climate change.

**Table 1:** The Espoo cohort study, 20-year follow-up 2010–2011: the understanding of and concern over climate change in different socio-demographic groups.

Characteristic	Understanding^a^			Concern^b^				Total n (%)
	Poor n (%)	Good n (%)	P value^c^	Low n (%)	Medium n (%)	High n (%)	P value^c^	
**Gender**			<.0001				<.0001	
Female	50 (29.6)	794 (56.2)		90 (33.3)	401 (52.7)	364 (64.0)		869 (53.5)
Male	119 (70.4)	620 (43.8)		180 (66.7)	359 (47.2)	205 (36.0)		754 (46.5)
**Age group (yr)**			0.7067				0.1587	
20–23	96 (56.8)	824 (58.3)		165 (61.1)	453 (59.6)	314 (55.2)		948 (58.4)
24–27	73 (43.2)	589 (41.7)		105 (38.9)	307 (40.9)	255 (44.8)		675 (41.6)
**Highest qualification**			0.1762				0.0104	
Comprehensive school degree	12 (7.1)	91 (6.5)		22 (8.2)	50 (6.6)	33 (5.8)		105 (6.5)
Upper secondary school degree	76 (45.0)	678 (48.1)		122 (45.2)	368 (48.5)	272 (48.0)		772 (47.6)
Vocational school degree	36 (21.3)	202 (14.3)		55 (20.4)	117 (15.4)	68 (12.0)		245 (15.1)
Upper secondary and vocational school degree	8 (4.7)	72 (5.1)		18 (6.7)	36 (4.7)	26 (4.6)		81 (5.0)
Higher vocational or academic degree	37 (21.9)	367 (26.0)		53 (19.6)	188 (24.8)	168 (29.6)		415 (25.7)
Missing information								5
**Occupation**			0.1611				<.0001	
Studying	84 (50.9)	768 (55.4)		125 (47.7)	407 (54.6)	325 (58.0)		861 (54.7)
Factory/mining/construction	17 (10.3)	107 (7.7)		41 (15.7)	53 (7.1)	30 (5.4)		124 (7.9)
Office/service	46 (27.9)	398 (28.7)		68 (26.0)	224 (30.1)	159 (28.4)		454 (28.8)
Stay-at-home mother/father	3 (1.82)	23 (1.66)		3 (1.2)	16 (2.2)	11 (2.0)		30 (1.9)
Unemployed	14 (8.5)	63 (4.55)		23 (8.8)	33 (4.4)	21 (3.8)		77 (4.9)
Other	1 (0.61)	27 (1.95)		2 (0.8)	12 (1.6)	14 (2.5)		28 (1.8)
Missing information								49
**Marital status**			0.1720				0.7435	
Single	116 (69.1)	866 (61.3)		173 (64.3)	460 (60.3)	365 (62.6)		1003 (62.0)
Married/civil partnership	9 (5.4)	70 (5.9)		14 (5.2)	41 (5.4)	26 (4.6)		82 (5.1)
Cohabitation	42 (25.0)	472 (33.4)		80 (29.7)	256 (33.7)	186 (32.7)		528 (32.6)
Divorced/separated	1 (0.60)	5 (0.35)		2 (0.7)	3 (0.39)	1 (0.2)		6 (0.4)
Missing information								4
**Children**								
No	161 (95.3)	1334 (94.6)	0.7194	252 (93.3)	714 (94.1)	542 (95.6)	0.3218	1518 (94.5)
Yes	8 (4.7)	76 (5.4)		18 (6.7)	45 (5.9)	25 (4.4)		88 (5.5)
Missing information								17
**Income (€/yr)**			0.7763				0.0297	
≤8400	62 (37.4)	484 (35.5)		88 (34.1)	267 (36.4)	198 (35.8)		556 (35.9)
8401–16800	59 (35.5)	474 (34.8)		92 (35.7)	231 (31.5)	216 (39.1)		541 (34.9)
≥16801	45 (27.1)	405 (29.7)		78 (30.2)	235 (32.1)	139 (25.1)		454 (29.3)
Missinginformation								72

a Missing information n = 41;

b missing information n = 24;

c the P value for X^2^ test.

### The Determinants of Climate-friendly Food Consumption

Respondents with good understanding of climate change reported to eat fresh vegetables/salad/root vegetables, fresh fruits, soy products, vegetable oils, organic food and rice more frequently and French fries and pork/beef/lamb less frequently than respondents with poor understanding (Table S1). Respondents highly concerned about climate change reported to eat vegetables/salad/root vegetables, fresh fruits, soy products, vegetable oils, organic food and low fat cheese more frequently and pork/beef/lamb and French fries less frequently (Table S2).

The average CFDS of the whole study population was 0.56 (SD 3.09). CFDS’s of the respondents ranged from −11 to 11. The people with good understanding of climate change had higher average CFDS (0.67, SD 3.11) than those with poor understanding (−0.46, SD 2.70). The average CFDS increased gradually as the concern over climate change increased: from −0.62 (low concern) to 0.37 (medium concern) to 1.37 (high concern) (Table 2).

**Table 2:** The Espoo cohort study, 20-year follow-up 2010–2011: linear regression analysis of CFDS against understanding of climate change, concern about climate change and socio-demographic variables.

	The whole study population (n = 1364)				Females (n = 726)			Males (n = 638)		
Determinant	Mean CFDS (SD)	Beta	95% CI	p value	Beta	95% CI	p value	Beta	95% CI	p value
**Understanding**										
Poor (ref)	−0.46 (2.70)									
Good	0.67 (3.11)	0.13	−0.40, 0.67	0.6264	0.79	−0.11, 1.70	0.0858	−0.18	−0.84, 0.49	0.6052
**Concern**										
Low (ref)	−0.62 (2.76)									
Medium	0.37 (3.00)	0.51	0.03, 0.98	0.0357	0.71	−0.08, 1.50	0.0766	0.42	−0.18, 1.03	0.1672
High	1.37 (3.15)	1.30	0.80, 1.80	<.0001	1.52	0.72, 2.32	0.0002	1.16	0.48, 1.83	0.0008
**Gender**										
Male (ref)	−0.54 (2.89)									
Female	1.50 (2.95)	1.92	1.59, 2.25	<.0001						
**Highest qualification**										
Comprehensive school (ref)	0.08 (2.98)									
Upper secondary school	0.71 (3.09)	0.50	−0.20, 1.20	0.1611	0.30	−0.68, 1.27	0.5478	0.63	−0.40, 1.66	0.2282
Vocational school	−0.21 (3.02)	0.02	−0.73, 0.78	0.9525	−0.12	−1.17, 0.94	0.8435	0.20	−0.91, 1.31	0.7239
Upper secondary and vocational school	0.42 (2.82)	0.11	−0.82, 1.04	0.8153	−0.35	1.59, 0.90	0.5874	0.85	−0.59, 2.28	0.2472
Higher vocational or academic	0.86 (3.14)	0.33	−0.39, 1.06	0.3651	0.18	-0.81, 1.16	0.7264	0.33	−0.78, 1.43	0.5617
**Occupation**										
Studying (ref)	0.79 (3.19)									
Factory/mining/construction	−0.56 (2.42)	−0.13	−0.80, 0.54	0.7015	−1.25	−2.88, 0.39	0.1347	0.08	−0.71, 0.88	0.8400
Office/service	0.53 (3.01)	−0.30	−0.73, 0.12	0.1599	−0.53	−1.10, 0.03	0.0654	−0.06	−0.71, 0.60	0.8653
Stay-at-home mother/father	0.89 (2.49)	−0.18	−1.61, 1.26	0.8063	−0.19	−1.79, 1.42	0.8188	a		
Unemployed	−0.54 (2.72)	−0.92	−1.68, −0.15	0.0186	−1.82	−3.01, −0.64	0.0026	−0.31	−1.32, 0.70	0.5447
Other	1.12 (3.14)	−0.18	−1.31, 0.95	0.7549	−0.77	−2.12, 0.58	0.2612	0.97	−1.17, 3.12	0.3717
**Marital status**										
Single (ref)	0.57 (3.09)									
Married/civil partnership	0.90 (2.57)	0.46	−0.31, 1.23	0.2448	0.42	−0.59, 1.42	0.4151	0.44	−0.80, 1.69	0.4864
Cohabitation	0.51 (3.18)	−0.20	−0.55, 0.15	0.2520	−0.15	−0.62, 0.32	0.5306	−0.27	−0.79, 0.25	0.3121
Divorced/separated	−1.00 (2.61)	−1.40	−3.75, 0.94	0.2409	−1.12	−3.77, 1.52	0.4043	−1.16	−6.80, 4.47	0.6854
**Children**										
No (ref)	0.58 (3.08)									
Yes	0.04 (3.04)	−0.35	−1.22, 0.51	0.4258	−0.59	−1.75, 0.57	0.3176	−0.07	−1.44, 1.29	0.9143
**Income (€/yr)**										
≤8400 (ref)	0.78 (3.18)									
8401–16800	0.53 (2.99)	−0.42	−0.80, −0.05	0.0272	−0.74	−1.26, −0.21	0.0054	0.00	−0.55, 0.54	0.9884
≥16801	0.30 (3.04)	−0.20	−0.66, 0.27	0.4059	−0.31	−0.97, 0.34	0.3486	−0.03	−0.70, 0.64	0.9265

a No data.

Multivariate linear regression analyses on the determinants of CFDS revealed that medium concern raised CFDS on average by 0.51 points (95% CI 0.03, 0.98) and high concern by 1.30 points (95% CI 0.80, 1.80) (Table 2) compared to low concern. This result is in accordance with the hypothesis that people concerned with climate change make climate-friendlier dietary choices. Unlike we hypothesized, understanding of climate change did not affect CFDS on its own. Female gender raised CFDS by 1.92 (95% CI 1.59, 2.25) when compared to males. This result was in line with the hypothesis. Unemployment decreased CFDS by 0.92 (95% CI −1.68, −0.15) when compared to studying and income in the medium range (€8,401–16,800/year) by 0.42 (95% CI −0.80, −0.05) when compared to the lowest income category. Education did not have an effect on CFDS, unlike we hypothesized.

When females and males were analyzed separately (Table 2), it could be seen that high concern over climate change brought about a greater increase in CFDS in females (1.52, 95% CI 0.72, 2.32) than in males (1.16, 95% CI 0.48, 1.83). The effect of medium concern weakened in the separate analyses of the genders. Among females, good understanding of climate change weakly increased CFDS (0.79, 95% CI −0.11, 1.70) compared to poor understanding. Unemployment decreased CFDS by 1.82 (95% CI −3.01, −0.64) and income in category €8,401–16,800/year by 0.74 (95% CI −1.26, −0.21) among females.

## Discussion

### Main Findings

Respondents highly concerned about climate change made climate-friendlier dietary choices than people who were only slightly or not at all concerned. The high concern over climate change had a greater effect on dietary choices among females than males. The level of understanding of climate change was only weakly connected to climate-friendly dietary choices in females but not in males. Unemployed females had less climate-friendly diets than females in other occupational groups.

### Validity of Results

The assessment of understanding of climate change was based on a question about the presumed causes of the phenomenon (see *Assessment of awareness of climate change*). Answer alternatives 3 and 4 were taken to indicate good understanding of climate change. As stated in alternative 4, anthropogenic GHG emissions are very likely the cause of the increase in global average temperatures [6]. Alternative 3 also indicates good understanding since it lists in a general way the human activities that cause these GHG emissions. Alternatives 1 and 2 indicate poor understanding since it is very unlikely that climate change is caused by known natural external causes alone [6]. Alternative 5 also indicates weaker understanding because there is increased confidence that natural internal variability cannot be the cause of the observed changes in the climate [6].

The assessment of the climate-friendliness of the food items was based on information about the GHG emission generated during their life cycles. In the case of French fries information on energy consumption was used [12] because information on GHG emissions was not available. However, the energy consumption during the production and processing of a food item is not a very accurate measure of the product’s climate-friendliness. This is because some GHGs emitted during the production of a food item may be non-energy related [9]. Organic food was categorized as climate-friendly but it is unclear whether organic food is always better for the climate than conventionally produced food. Organic farming increases carbon sequestration in soil [32] and generates less nitrous oxide emissions than conventional farming [8] but tends to have higher CO_2_ emissions on per-unit output scale [34]. We classified fresh vegetables as climate-friendly food, even though they are not very climate-friendly if they are grown in heated greenhouses [31]. However, the majority of Finnish vegetables are grown in open fields (66.4% in 2012) [35], which causes very little GHG emissions [30]. Thus classifying fresh vegetables as climate-friendly is minimal erroneous.

One of the strengths of our study is that we were able to assess the consumption frequencies of a wide variety of food items. However, CFDS could be further improved to include information on consumption of local food products and avoiding food waste, since these food-related behaviors are relevant in climate change mitigation [12], [36].

We compared the food consumption frequencies of our study population to the consumption frequencies reported in a national study of Finnish adults (aged 25–74 years) [13]. Our study population and the general Finnish adult population had the same median intake frequencies of rice, low fat cheese, other cheese, boiled or mashed potatoes, French fries, fresh vegetables/salad, poultry and red meat. This indicates that the dietary choices of our study population did not seem to substantially diverge from those of the general Finnish adult population.

We did not have information on the actual amounts (in e.g. grams) of the foods consumed by the study subjects. Only the intake frequencies could be taken into account when calculating the CFDS. Therefore the CFDS is not an absolute measure of the GHGs caused by a person’s diet. Rather, CFDS helps to evaluate if a person has a tendency to consume climate-friendly food items. Therefore CFDS gives an approximation of the climate-friendliness of the dietary choices on the whole. Some food items such as cheese had a slightly pronounced influence on CFDS because we gave points on the intake frequency of low fat cheese and other types of cheese separately. On the other hand, we handled the red meats (pork/beef/lamb) causing huge GHG emissions as a single food choice. Therefore the choice of consuming several types of red meat might have influenced the CFDS too little.

The cohort members had to answer the dietary questions on the basis of their food consumption during the past 12 months. This is a potential source of information bias since it might have been hard for the cohort members to remember precisely their food consumption patterns for the past year. However, the possible error most probably is not systematic and therefore this it is not likely that the CFDSs are biased to any specific direction.

There are plenty of factors affecting dietary choices and we were able to take many of them into account. Age, sex, marital status, parental status, occupation, education, and income level affect food behavior [37], [38]. In the present study the study subjects had a narrow age range (20 to 27 years) and the other above mentioned factors were included in the regression analyses. However, there are several psychological factors that may influence climate-friendly food choices that were not taken into account in our study. For example a recent study by Dowd and Burke (2013) indicates that positive moral attitude and ethical concern predict the intention to purchase sustainably sourced foods [39]. These kinds of factors may also affect the intention to consume and the actual consumption of climate-friendly food. In addition, because climate-friendly eating may delay or avert death of chronic diseases [4] health reasons rather than environmental reasons might be behind the climate-friendly food choices. Indicator of this is the fact that the highly concerned in our study population ate more frequently low-fat cheese, which is healthier than normal cheese but equally harmful to the climate. Personal preferences such as familiarity and sensory appeal may also play a role in food choice [40]. However, we believe that CFDS is not prone to error from such sources because CFDS is mostly based on the consumption of food categories (e.g. fresh fruits) rather than specific food products. Therefore there is room for personal preferences inside many of the food categories incorporated to CFDS.

There is a possibility of selection bias because all the cohort members did not answer the questionnaire (the response rate being 64.0%). The theme of the questionnaire was climate change, environment and health. Thus people especially interested in environmental issues might have been more eager to answer the questionnaire. This seems not to be the case with this study, because 35.6% of our sample was highly concerned about climate change whereas Eurobarometer 2008 found 78% of Finnish people to be highly concerned (both measured on a 3-point scale) [16]. This remarkable difference between the percentages might be due to different age groups studied or different study times.

It can be regarded as a weakness of our study that we did not ask the respondents if they are aware of the link between food consumption and climate change. However, according to a national study on the climate change perceptions of Finnish people, 42% of Finnish adults agree that favoring a plant-based diet has quite a big effect or a big effect on climate change [17]. Hence we argue that it is not a remarkable weakness of the present study that we assume Finnish people to recognize the link between dietary choices and climate change.

### Synthesis with Previous Knowledge

There are only a few previous studies on the predictors of climate-friendly dietary choices. Mäkiniemi and Vainio (2013) found that the perceived seriousness of climate change consequences predicted climate-friendly food choices in their study population, that consisted of university students in the social and behavioral sciences of whom 80% were female [18]. Mäkiniemi’s and Vainio’s construct “Probable Seriousness of Consequences” is quite similar to the variable “concern” in our study: both of the measures aim to assess how a great threat climate change is perceived to be by the respondent. Because our study population was more representative of the general population (in terms of occupations, educational backgrounds and gender), our results add to the previous knowledge. In our study, too, the concern was connected to climate-friendlier diet. But we also found that this effect is stronger among females and that there are some special socio-demographic groups, such as unemployed females, that do not tend to make climate-friendly food choices.

It has previously been found that intention to change food consumption in order to mitigate climate change increases with worry about climate change consequences [41]. We did not study intentions but assessed the actual food intake frequencies. Therefore our study adds to the previous knowledge: the high concern about climate change might actually concretize the intentions to make dietary adjustments.

Other climate change mitigation behaviors and the factors affecting those behaviors have been studied more widely. In accordance with those studies our results indicate that high concern over climate change [20], [21] and female gender [24], [25] are strong predictors of climate change mitigation action.

It has been argued that when it comes to mitigating climate change people do not usually act in accordance with what they know or care about (the knowledge-action gap or the value-action gap) [19], [23]. Our results are partially in line with this argument. We discovered that understanding of climate change was only weakly connected to the dietary choices. But then again high concern over climate change was clearly connected to climate-friendlier food consumption. This result is understandable since large majority of the respondents (89.3%) had a good understanding over climate change: the topic is widely discussed in the media and schools in Finland. Information about climate change is hence easily accessible without hard personal effort. Concern, on the other hand, requires more active personal reflection. Therefore it is understandable that concern over climate change has a more remarkable effect on the food choices.

A diet that is climate-friendly is likely to be healthier. Many studies support the fact that decreasing red meat consumption would reduce GHG emissions and the risk for several chronic diseases simultaneously. A study by Scarborough et al. found that reduction in meat and dairy consumption replaced by vegetables, fruit and cereals averts deaths from coronary heart disease, stroke and cancer as well as reduces GHG emissions [4]. Aston et al. (2012) calculated that a reduction in red meat and processed meat intake would remarkably decrease GHG emissions and the incidence of coronary heart disease, diabetes mellitus and colorectal cancer [3]. Friel et al. (2009) conclude that decreased livestock production would reduce GHG emissions and decrease deaths and disability caused by ischemic heart disease [5]. Thus adjusting into climate-friendlier diet can have positive effects on public health. This aspect should be more clearly emphasized when promoting climate-friendly lifestyles.

## Conclusions

In this study among young Finnish adults, concern about climate change was connected to climate-friendly food choices among both genders. The level of understanding of climate change was only weakly connected to climate-friendly dietary choices among females but not among males. Our results indicate that increasing awareness of climate change could lead to increased use of climate-friendly food items, reduction in GHG emissions, and thus climate change mitigation.

## Supporting Information

Table S1 **Understanding of climate change and the consumption frequencies of selected food items.**
(DOC)

Table S2 **Concern over climate change and the consumption frequencies of selected food items.**
(DOC)
