# Supplementary material for: Awareness of Climate Change and the Dietary Choices of Young Adults in Finland: A Population-Based Cross-Sectional Study
Source: PLoS One. 2014 May 13;9(5):e97480. doi: 10.1371/journal.pone.0097480 (PMC4019576; doi:10.1371/journal.pone.0097480)
Supplement: Table S1 — Understanding of climate change and the consumption frequencies of selected food items. (DOC) [file pone.0097480.s001.doc]

Table S1. The Espoo cohort study, 20-year follow-up 2010-2011: understanding of climate change and the consumption frequencies of selected food items (*Italics* = median intake frequency in the study population).

|  | Less than once a month n(%) | 1-3 times a month n(%) | 1-3 times a week n(%) | Almost daily n(%) | At least once a day n(%) | Total n(%) | P for trend |
| --- | --- | --- | --- | --- | --- | --- | --- |
| **French fries** |  |  |  |  |  |  | 0.0075 |
| Poor understanding | 36 (7.8) | *92 (12.0)* | 31 (10.0) | 7 (31.8) | 1 (25.0) | 167 (10.7) |  |
| Good understanding | 425 (92.2) | *678 (88.1)* | 277 (89.9) | 15 (68.2) | 3 (75.0) | 1398 (89.3) |  |
| Missing information |  |  |  |  |  | 58 |  |
| **Rice** |  |  |  |  |  |  | 0.0428 |
| Poor understanding | 12 (12.9) | 55 (12.7) | *82 (9.5)* | 18 (10.3) | 0 (0.0) | 167 (10.6) |  |
| Good understanding | 81 (87.1) | 377 (87.3) | *780 (90.5)* | 156 (89.7) | 10 (100.0) | 1404 (89.4) |  |
| Missing information |  |  |  |  |  | 52 |  |
| **Pork/beef/lamb** |  |  |  |  |  |  | 0.0017 |
| Poor understanding | 9 (5.3) | 24 (9.8) | *99 (11.0)* | 31 (15.4) | 3 (12.0) | 166 (10.8) |  |
| Good understanding | 161 (94.7) | 221 (90.2) | *804 (89.0)* | 170 (84.6) | 22 (88.0) | 1378 (89.3) |  |
| Missing information |  |  |  |  |  | 79 |  |
| **Poultry** |  |  |  |  |  |  | 0.2190 |
| Poor understanding | 25 (11.3) | 33 (13.7) | *86 (9.6)* | 20 (11.8) | 2 (10.0) | 166 (10.7) |  |
| Good understanding | 196 (88.7) | 208 (86.3) | *814 (90.4)* | 150 (88.2) | 18 (90.0) | 1386 (89.3) |  |
| Missing information |  |  |  |  |  | 71 |  |
| **Low fat cheese** |  |  |  |  |  |  | 0.0722 |
| Poor understanding | 44 (11.6) | 33 (11.5) | *36 (9.6)* | 38 (11.8) | 12 (6.3) | 163 (10.5) |  |
| Good understanding | 334 (88.4) | 253 (88.5) | *339 (90.4)* | 284 (88.2) | 180 (93.8) | 1390 (89.5) |  |
| Missing information |  |  |  |  |  | 70 |  |
| **Other cheese** |  |  |  |  |  |  | 0.3320 |
| Poor understanding | 41 (10.8) | *50 (11.4)* | 37 (9.4) | 24 (10.0) | 11 (11.0) | 163 (10.5) |  |
| Good understanding | 339 (89.2) | *387 (88.6)* | 357 (90.6) | 215 (90.0) | 89 (89.0) | 1387 (89.5) |  |
| Missing information |  |  |  |  |  | 73 |  |
| **Butter** |  |  |  |  |  |  | 0.1179 |
| Poor understanding | *91 (10.1)* | 42 (10.6) | 21 (11.5) | 8 (16.3) | 3 (12.5) | 165 (10.6) |  |
| Good understanding | *809 (89.9)* | 353 (89.4) | 162 (88.5) | 41 (83.7) | 21 (87.5) | 1386 (89.4) |  |
| Missing information |  |  |  |  |  | 72 |  |
| **Potato, boiled/mashed** |  |  |  |  |  |  | 0.4230 |
| Poor understanding | 19 (15.0) | 42 (10.6) | *78 (9.4)* | 20 (10.8) | 8 (22.9) | 167 (10.6) |  |
| Good understanding | 108 (85.0) | 354 (89.4) | *750 (90.6)* | 165 (89.1) | 27 (77.1) | 1404 (89.4) |  |
| Missing information |  |  |  |  |  | 52 |  |
| **Fresh vegetables/ root vegetables/ salad** |  |  |  |  |  |  | <.0001 |
| Poor understanding | 10 (28.6) | 28 (20.3) | 44 (10.8) | *56 (10.3)* | 29 (6.5) | 167 (10.6) |  |
| Good understanding | 25 (71.4) | 110 (79.7) | 362 (89.2) | *488 (89.7)* | 416 (93.5) | 1401 (89.4) |  |
| Missing information |  |  |  |  |  | 55 |  |
| **Fresh fruits** |  |  |  |  |  |  | <.0001 |
| Poor understanding | 9 (11.0) | 46 (18.0) | *63 (11.3)* | 29 (7.5) | 20 (7.2) | 167 (10.7) |  |
| Good understanding | 73 (89.0) | 209 (82.0) | *494 (88.7)* | 358 (92.5) | 258 (92.8) | 1392 (89.3) |  |
| Missing information |  |  |  |  |  | 64 |  |
| **Soy products** |  |  |  |  |  |  | 0.0014 |
| Poor understanding | *133 (12.2)* | 26 (9.8) | 4 (2.92) | 3 (6.8) | 1 (8.33) | 167 (10.8) |  |
| Good understanding | *961 (87.8)* | 239 (90.2) | 133 (97.1) | 41 (93.2) | 11 (91.7) | 1385 (89.2) |  |
| Missing information |  |  |  |  |  | 71 |  |
| **Vegetable oils** |  |  |  |  |  |  | 0.0083 |
| Poor understanding | 33 (13.2) | 39 (14.9) | *50 (9.0)* | 34 (9.4) | 10 (8.26) | 166 (10.7) |  |
| Good understanding | 218 (86.9) | 223 (85.1) | *506 (91.0)* | 328 (90.6) | 111 (91.7) | 1386 (89.3) |  |
| Missing information |  |  |  |  |  | 71 |  |
| **Margarine** |  |  |  |  |  |  | 0.2469 |
| Poor understanding | 39 (10.7) | 25 (11.8) | *27 (10.0)* | 41 (12.5) | 33 (8.6) | 165 (10.6) |  |
| Good understanding | 326 (89.3) | 187 (88.2) | *244 (90.0)* | 287 (87.5) | 352 (91.4) | 1396 (89.4) |  |
| Missing information |  |  |  |  |  | 62 |  |
| **Organic food** | Not at all | Less than once a month | 1-3 days a month | 1-3 days a week | daily or almost daily |  | 0.0005 |
| Poor understanding | 68 (16.0) | *38 (8.8)* | 30 (8.8) | 14 (5.93) | 14 (10.7) | 164 (10.5) |  |
| Good understanding | 356 (84.0) | *396 (91.2)* | 312 (91.2) | 222 (94.1) | 117 (89.3) | 1403 (89.5) |  |
| Missing information |  |  |  |  |  | 56 |  |
