# Supplementary material for: Awareness of Climate Change and the Dietary Choices of Young Adults in Finland: A Population-Based Cross-Sectional Study
Source: PLoS One. 2014 May 13;9(5):e97480. doi: 10.1371/journal.pone.0097480 (PMC4019576; doi:10.1371/journal.pone.0097480)
Supplement: Table S2 — Concern over climate change and the consumption frequencies of selected food items. (DOC) [file pone.0097480.s002.doc]

Table S2. The Espoo cohort study, 20-year follow-up 2010-2011: concern over climate change and the consumption frequencies of selected food items (*Italics* = median intake frequency in the study population).

|  | Less than once a month n(%) | 1-3 times a month n(%) | 1-3 times a week n(%) | Almost daily n(%) | At least once a day n(%) | Total n(%) | P for trend^a^ |
| --- | --- | --- | --- | --- | --- | --- | --- |
| **French fries** |  |  |  |  |  |  | <.0001 |
| Low concern | 56 (12.0) | *137 (17.6)* | 63 (20.1) | 8 (36.4) | 2 (50.0) | 266 (16.8) |  |
| Medium concern | 212 (45.6) | *385 (49.5)* | 145 (46.3) | 7 (31.8) | 1 (25.0) | 750 (47.4) |  |
| High concern | 197 (42.4) | *256 (32.9)* | 105 (33.6) | 7 (31.8) | 1 (25.0) | 566 (35.8) |  |
| Missing information |  |  |  |  |  | 41 |  |
| **Rice** |  |  |  |  |  |  | 0.4506 |
| Low concern | 15 (15.5) | 74 (17.1) | *152 (17.4)* | 25 (14.3) | 0 (0.0) | 266 (16.8) |  |
| Medium concern | 41 (42.3) | 207 (47.7) | *411 (47.1)* | 88 (50.3) | 6 (60.0) | 753 (47.4) |  |
| High concern | 41 (42.3) | 153 (35.3) | *309 (35.4)* | 62 (35.4) | 4 (40.0) | 569 (35.8) |  |
| Missing information |  |  |  |  |  | 35 |  |
| **Pork/beef/lamb** |  |  |  |  |  |  | <.0001 |
| Low concern | 16 (9.2) | 35 (14.0) | *161 (17.7)* | 45 (22.2) | 8 (32.0) | 265 (17.0) |  |
| Medium concern | 64 (36.8) | 117 (46.8) | *440 (48.5)* | 105 (51.7) | 12 (48.0) | 738 (47.3) |  |
| High concern | 94 (54.0) | 98 (39.2) | *307 (33.8)* | 53 (26.1) | 5 (20.0) | 557 (35.7) |  |
| Missing information |  |  |  |  |  | 63 |  |
| **Poultry** |  |  |  |  |  |  | 0.1332 |
| Low concern | 32 (14.1) | 54 (22.3) | *154 (17.0)* | 21 (12.1) | 4 (20.0) | 265 (16.9) |  |
| Medium concern | 88 (38.9) | 105 (43.4) | *439 (48.4)* | 106 (60.9) | 7 (35.0) | 745 (47.5) |  |
| High concern | 106 (46.9) | 83 (34.3) | *314 (34.6)* | 47 (27.0) | 9 (45.0) | 559 (35.6) |  |
| Missing information |  |  |  |  |  | 54 |  |
| **Low fat cheese** |  |  |  |  |  |  | 0.0089 |
| Low concern | 75 (19.5) | 55 (19.3) | *54 (14.3)* | 52 (16.0) | 26 (13.4) | 262 (16.7) |  |
| Medium concern | 184 (47.8) | 128 (44.9) | *185 (48.8)* | 158 (48.5) | 90 (46.4) | 745 (47.5) |  |
| High concern | 126 (32.7) | 102 (35.8) | *140 (36.9)* | 116 (35.6) | 78 (40.2) | 562 (35.8) |  |
| Missing information |  |  |  |  |  | 54 |  |
| **Other cheese** |  |  |  |  |  |  | 0.3455 |
| Low concern | 61 (15.7) | *70 (15.9)* | 71 (17.9) | 40 (16.6) | 21 (21.0) | 263 (16.8) |  |
| Medium concern | 194 (49.9) | *221 (50.1)* | 170 (42.9) | 125 (51.9) | 35 (35.0) | 745 (47.5) |  |
| High concern | 134 (34.5) | *150 (34.0)* | 155 (39.1) | 76 (31.5) | 44 (44.0) | 559 (35.7) |  |
| Missing information |  |  |  |  |  | 56 |  |
| **Butter** |  |  |  |  |  |  | 0.1257 |
| Low concern | *150 (16.5)* | 68 (17.0) | 31 (16.8) | 9 (18.0) | 7 (29.2) | 265 (16.9) |  |
| Medium concern | *433 (47.6)* | 189 (47.3) | 87 (47.0) | 26 (52.0) | 11 (45.8) | 746 (47.6) |  |
| High concern | *327 (35.9)* | 143 (35.8) | 67 (36.2) | 15 (30.0) | 6 (25.0) | 558 (35.6) |  |
| Missing information |  |  |  |  |  | 54 |  |
| **Potato, boiled/mashed** |  |  |  |  |  |  | 0.4716 |
| Low concern | 25 (19.5) | 67 (16.9) | *130 (15.5)* | 33 (17.6) | 12 (34.3) | 267 (16.8) |  |
| Medium concern | 60 (46.9) | 198 (50.0) | *392 (46.6)* | 94 (50.0) | 10 (28.6) | 754 (47.5) |  |
| High concern | 43 (33.6) | 131 (33.1) | *319 (37.9)* | 61 (32.5) | 13 (37.1) | 567 (35.7) |  |
| Missing information |  |  |  |  |  | 35 |  |
| **Fresh vegetables/ root vegetables/salad** |  |  |  |  |  |  | <.0001 |
| Low concern | 8 (22.9) | 41 (29.5) | 89 (21.6) | *85 (15.5)* | 43 (9.6) | 266 (16.8) |  |
| Medium concern | 21 (60.0) | 67 (48.2) | 186 (45.2) | *276 (50.2)* | 203 (45.2) | 753 (47.5) |  |
| High concern | 6 (17.1) | 31 (22.3) | 137 (33.3) | *189 (34.4)* | 203 (45.2) | 566 (35.7) |  |
| Missing information |  |  |  |  |  | 38 |  |
| **Fresh fruits** |  |  |  |  |  |  | <.0001 |
| Low concern | 19 (22.9) | 69 (27.1) | *108 (19.2)* | 45 (11.5) | 26 (9.2) | 267 (16.9) |  |
| Medium concern | 36 (43.4) | 115 (45.1) | *263 (46.7)* | 202 (51.4) | 127 (44.9) | 743 (47.1) |  |
| High concern | 28 (33.7) | 71 (27.8) | *192 (34.1)* | 145 (37.1) | 130 (45.9) | 566 (35.9) |  |
| Missing information |  |  |  |  |  | 47 |  |
| **Soy products** |  |  |  |  |  |  | <.0001 |
| Low concern | *210 (19.0)* | 32 (12.0) | 14 (10.1) | 8 (17.0) | 1 (8.3) | 265 (16.9) |  |
| Medium concern | *556 (50.4)* | 118 (44.4) | 48 (34.5) | 14 (29.8) | 4 (33.3) | 740 (47.2) |  |
| High concern | *338 (30.6)* | 116 (43.6) | 77 (55.4) | 25 (53.2) | 7 (58.3) | 563 (35.9) |  |
| Missing information |  |  |  |  |  | 55 |  |
| **Vegetable oils** |  |  |  |  |  |  | <.0001 |
| Low concern | 63 (24.7) | 50 (19.0) | *87 (15.5)* | 43 (11.8) | 18 (14.5) | 261 (16.6) |  |
| Medium concern | 127 (49.8) | 127 (48.3) | *271 (48.3)* | 168 (45.9) | 53 (42.7) | 746 (47.6) |  |
| High concern | 65 (25.5) | 86 (32.7) | *203 (36.2)* | 155 (42.4) | 53 (42.7) | 562 (35.8) |  |
| Missing information |  |  |  |  |  | 54 |  |
| **Margarine** |  |  |  |  |  |  | 0.1803 |
| Low concern | 56 (15.3) | 39 (18.3) | *48 (17.8)* | 56 (16.7) | 65 (16.6) | 264 (16.7) |  |
| Medium concern | 163 (44.4) | 108 (50.7) | *119 (44.1)* | 163 (48.5) | 196 (50.0) | 749 (47.5) |  |
| High concern | 148 (40.3) | 66 (31.0) | *103 (38.2)* | 117 (34.8) | 131 (33.4) | 565 (35.8) |  |
| Missing information |  |  |  |  |  | 45 |  |
| **Organic food** | Not at all | <once a month | 1-3 days a month | 1-3 days a week | daily/almost daily |  | <.0001 |
| Low concern | 109 (25.3) | *64 (14.7)* | 51 (14.8) | 26 (10.9) | 13 (9.7) | 263 (16.6) |  |
| Medium concern | 222 (51.5) | *233 (53.3)* | 146 (42.4) | 108 (45.4) | 46 (34.3) | 755 (47.7) |  |
| High concern | 100 (23.2) | *140 (32.0)* | 147 (42.7) | 104 (43.7) | 75 (56.0) | 566 (35.7) |  |
| Missing information |  |  |  |  |  | 39 |  |

^a^Comparing low and high concern categories
